# A Ready Method for Removing Foreign Bodies from the Anterior Nares

**Published:** 1888-02

**Authors:** 


### *A READY METHOD FOR REMOVING FOREIGN BODIES FROM THE ANTERIOR NARES.*

Physicians are often called to remove peas, buttons, and various substances from the nostrils of children who have themselves introduced them there. A ready method for removing such substances is described by Mr. T. Osborne-Walker in the *Lancet* for Sept. 17, 1887, where he states that recently a little boy was brought under his care with a button tightly impacted in the angle between the vomer and os nasi at the bridge in the right nostril. Ineffectual attempts at extraction had evidently been made, as shown by blood oozing from the nostril, and some, coagulated, adherent to the button, partially concealing its outlines from view, and also by the button being fixedly jammed in. In such cases, to prevent struggles and interruption, the child's arms, hands and legs should be first confined, by folding tightly round these and the body a long, clean apron, and then placing the child on an attendant's lap, facing a window, while the operator stands behind the patient, and, bending over and depressing with two fingers of the left hand the apex of the nose, to admit as much light as possible upon the object to be removed, with the right hand very carefully, to avoid its descent into the pharynx or larynx, the spoon end (with the concavity directed forward) of an ordinary pocket-case director should be introduced, with which at once, with a simple lever movement or jerk, the foreign body may be readily ejected.

By attention to the following points, the removal is instantaneously effected. The close confinement of the hands, arms and legs by a shawl, blanket, or apron; a good light; a reliable person to securely hold the child; the position of the operator behind the patient; depressing well the apex of the nose to obtain a good view of the object; and, lastly, getting the concave face of the spoon of a director fairly behind the body before making the forward lever movement. —*Therapeutic Gazette.*

